# The CANVAS Program: implications of canagliflozin on reducing cardiovascular risk in patients with type 2 diabetes mellitus

**DOI:** 10.1186/s12933-019-0869-2

**Published:** 2019-05-28

**Authors:** Salvatore Carbone, Dave L. Dixon

**Affiliations:** 1 Department of Internal Medicine, Pauley Heart Center, Virginia Commonwealth University, West Hospital, Room 529b, 1200 E Broad Street, Richmond, VA 23298 USA; 2 Department of Pharmacotherapy and Outcome Sciences, School of Pharmacy, Virginia Commonwealth University, 410 N. 12th Street, Richmond, VA 23298 USA

**Keywords:** Diabetes mellitus, Major adverse cardiovascular event, Sodium glucose co-transporter 2 inhibitor, Canagliflozin

## Abstract

Canagliflozin is a sodium glucose co-transporter 2 (SGLT2) inhibitor that reduces blood glucose, as well as blood pressure, body weight, and albuminuria in patients with type 2 diabetes mellitus (T2DM). In the CANagliflozin cardioVascular Assessment Study (CANVAS) Program, patients with T2DM and high cardiovascular risk treated with canagliflozin had a significantly lower risk of the composite outcome of cardiovascular death, nonfatal myocardial infarction, or nonfatal stroke; hospitalization for heart failure; and renal outcomes, but also a greater risk of lower-limb amputation. Cardiovascular outcomes trials of some other T2DM agents (i.e., empagliflozin, dapagliflozin, liraglutide, semaglutide, albiglutide) have also shown potential cardiovascular and renal benefits. As a result, diabetes treatment guidelines have begun to incorporate consideration of cardiovascular and renal benefits into their treatment recommendations. Antihyperglycemic agents with proven beneficial cardiovascular effects represent a new opportunity for the diabetologist and cardiologist, in the setting of a multidisciplinary approach, to concomitantly improve glycemic control and reduce the risk of cardiovascular events in patients with T2DM. This review briefly discusses the pharmacology of canagliflozin, including clinical and preclinical data; it also describes the effects of canagliflozin on cardiovascular outcomes and side-effects, and compares these effects with other glucose-lowering agents with proven cardiovascular benefits.

## Introduction

In 2013, canagliflozin became the first sodium glucose co-transporter 2 (SGLT2) inhibitor approved by the US Food and Drug Administration (FDA) for reducing blood glucose in patients with type 2 diabetes mellitus (T2DM) as an adjunct to diet and exercise [1]. Approval was obtained based on the beneficial effects of canagliflozin on glycemic control, measured as a reduction in glycated hemoglobin (HbA1c), evaluated in 9 phase III clinical trials with over 10,000 patients [2–14]. Across these studies, canagliflozin was also associated with weight loss and blood pressure reduction [1]. The CANagliflozin cardioVascular Assessment Study (CANVAS) Program, consisting of the CANVAS study and CANVAS-R (renal), cardiovascular outcomes trials (CVOTs) assessed the cardiovascular (CV) safety and efficacy of canagliflozin in patients with T2DM and established cardiovascular disease (CVD) or at least 2 risk factors for CVD [15]. The CANVAS Program results demonstrated benefits of reduced risk of a composite outcome of major adverse cardiovascular events (MACE; CV death, nonfatal myocardial infarction [MI], or nonfatal stroke), but also a greater risk of lower-limb amputation, an overall low frequency event, with canagliflozin versus placebo [16]. CVOTs of the SGLT2 inhibitors empagliflozin and dapagliflozin and the glucagon-like peptide-1 (GLP-1) receptor agonists liraglutide, semaglutide, and albiglutide have also shown CV benefits [17–19].

In this review, we aim to briefly summarize canagliflozin pharmacology and clinical trial results and describe the CANVAS Program and other CVOTs and implications for cardiologists.

## Canagliflozin: pharmacology and results of phase III clinical trials

**Fig. 1:**
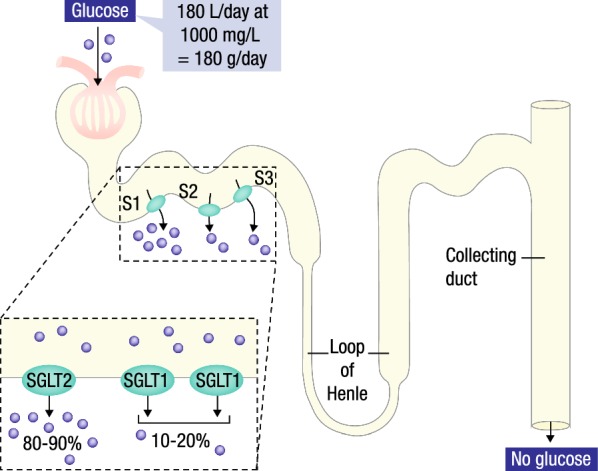
Renal actions of SGLT2 inhibitors. SGLT2, sodium glucose co-transporter 2; SGLT1, sodium glucose co-transporter 1 (Modified with permission from De Fronzo et al. [21])

The SGLT gene family encodes membrane proteins that regulate the transport of glucose, amino acids, vitamins, and ions [20]. They are mostly located in the proximal renal tubules, in the gut epithelium, and more recently were found in the heart [20]. SGLT2 is primarily located at the first segment of the proximal tubule level of the kidney and is responsible for about 90% of glucose reabsorption at the glomerulus level [21]. The remaining glucose is reabsorbed by SGLT1, which is located in the more distal segment of the tubule (Fig. 1) [21].

**Fig. 2:**
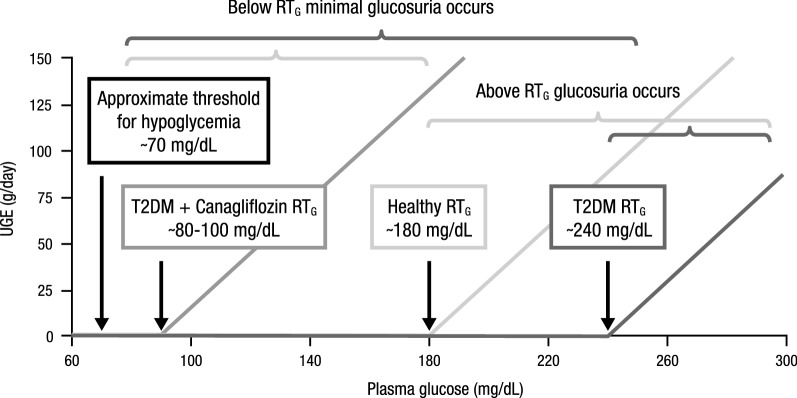
UGE with canagliflozin. UGE, urinary glucose excretion; RT_G_, renal threshold for glucose; T2DM, type 2 diabetes mellitus (Modified with permission from Wilding [22])

SGLT2 inhibitors reduce the renal threshold for glucose (RT_G_) and increase urinary glucose excretion (UGE) resulting in improved glycemic control (Fig. 2). In healthy individuals, RT_G_ is approximately 180 mg/dL; however, RT_G_ is significantly higher in patients with T2DM, at approximately 240 mg/dL, and is a primary contributor to chronic hyperglycemia [22]. The higher RT_G_ seen in patients with T2DM seems to result from increased renal SGLT2 expression; therefore, inhibition of SGLT2 represents an efficacious glucose-lowering therapeutic strategy [22]. In patients with T2DM, SGLT2 inhibitors typically lower RT_G_ to 70–90 mg/dL and increase UGE by 60–100 g/day [22]. Importantly, SGLT2 inhibitors improve glycemic control via an insulin-independent mechanism that is unique among antihyperglycemic agents, which primarily act on insulin secretion or insulin sensitization.

Canagliflozin is orally administered and rapidly absorbed with a bioavailability of 65%; peak plasma concentrations are reached within 1–2 h post-administration and steady state concentrations are reached after about 4 days [23]. Canagliflozin may be dosed 100 or 300 mg once daily; patients with estimated glomerular filtration rate (eGFR) < 60 mL/min/1.73 m^2^ are limited to receiving the 100 mg dose of canagliflozin and canagliflozin is not recommended for patients with eGFR < 45 mL/min/1.73 m^2^ [24]. In patients with T2DM, canagliflozin dose-dependently lowers RT_G_ to 70–90 mg/dL [25, 26].

Phase III clinical trials demonstrated significant reductions in HbA1c of 0.5–1.0% as well as reductions in body weight and blood pressure with canagliflozin compared with placebo [1]. In addition to reducing body weight and blood pressure, canagliflozin improved several other CV risk factors, such as body composition, uric acid levels, vascular stiffness, pulse pressure, cardiac work load, and magnesium levels [27–30]. Of note, such effects were reported in both patients without history of CVD and with established CVD [31]. Modest increases in low-density lipoprotein (LDL) cholesterol were also observed [1]. Common adverse effects observed in phase III clinical trials of canagliflozin were consistent with the SGLT2 inhibitor mechanism of action and included genital mycotic infections and osmotic diuresis-related adverse events [1]. The risk of hypoglycemic events with canagliflozin was extremely low in phase III clinical trials, and was most often associated with the use of background insulin or insulin secretagogues (e.g., sulfonylureas) [1]; this low risk of hypoglycemic events is desirable, considering hypoglycemia is associated with higher CV risk [32].

Preclinical data has also suggested that canagliflozin may reduce infarct size [33] as well as the progression of atherosclerosis, adhesion molecules, and markers of inflammation (i.e., vascular cell adhesion molecule-1 and monocyte chemotaxis protein-1) [34]. Furthermore, canagliflozin has shown potential beneficial effects on cardiac function. Particularly, canagliflozin improved cardiac diastolic function in patients with T2DM [35]. Furthermore, canagliflozin has been associated with a delay rise in biomarkers of cardiac wall stress (i.e., N-terminal pro-B-type natriuretic peptide and high-sensitivity troponin I) as well as an increase in hematocrit [36], consistent with what has been reported with other SGLT2 inhibitors [37].

## The CANVAS Program

### Design

The CANVAS Program is comprised of the integrated analysis of 2 similarly designed and conducted CVOTs, the CANVAS study and CANVAS-R. The history, design, and integrated analysis plan for data from the CANVAS Program has previously been described [15, 38, 39]. Briefly, the primary goal of the CANVAS Program was to demonstrate the safety of canagliflozin on MACE (CV death, nonfatal MI, or nonfatal stroke) compared with placebo on the background of standard of care for cardiovascular and diabetes risk factors [15]. The primary hypothesis was a test of noninferiority for the hazard ratio (HR) for MACE comparing pooled canagliflozin doses versus placebo using the full integrated dataset; CV safety would be demonstrated if the upper bound of the 95% confidence interval (CI) for the HR was < 1.3 and superiority would be demonstrated if the upper bound of the 95% CI for the HR was < 1.0 [15]. Statistical testing of the secondary hypotheses for all-cause mortality and CV-specific mortality, using the truncated integrated dataset that excluded the CANVAS study time and mortality events accrued prior to November 20, 2012, was planned to proceed sequentially if the primary hypothesis was met [15].

The CANVAS Program enrolled 10,142 patients with T2DM (HbA1c ≥ 7.0% and ≤ 10.5%) and established CVD or at least 2 risk factors for CVD from 30 countries (667 sites) who were followed for a mean of 3.6 years and maximum of 6.5 years [16]. After a 2-week placebo run-in period, patients were randomized to canagliflozin 100 mg, canagliflozin 300 mg, or placebo in the CANVAS study (4330 patients), while in CANVAS-R, patients were randomized to canagliflozin 100 mg, with the option of increasing to 300 mg after 13 weeks of treatment, or placebo (5812 patients) [38, 39]. The primary prevention cohort included 3486 (34%) patients who were 50 years or older with at least 2 CV risk factors, while the secondary prevention cohort included 6656 (66%) patients who were 30 years or older with symptomatic atherosclerotic CVD [16, 40]. Participants were required to have an eGFR ≥ 30 mL/min/1.73 m^2^ for enrollment.

### Effects of canagliflozin on CV risk factors in the CANVAS Program

**Fig. 3:**
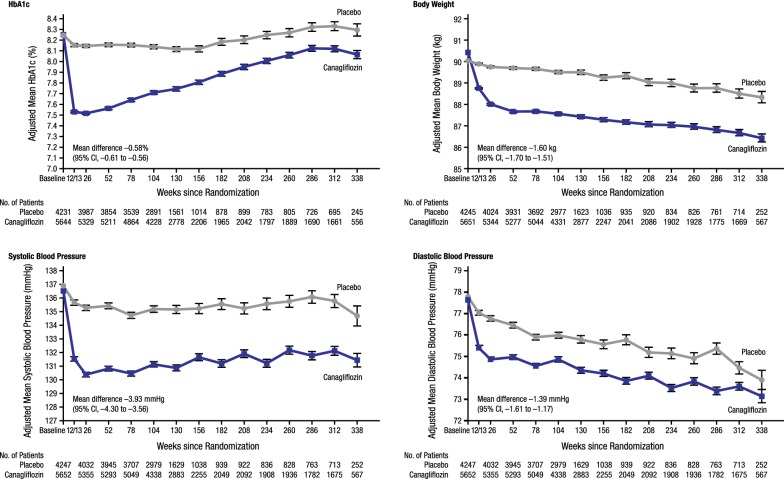
The CANVAS Program: effects of canagliflozin on HbA1c, body weight, systolic and diastolic blood pressure. HbA1c, glycated hemoglobin; CI, confidence interval (Reprinted with permission from Neal et al. [16])

Canagliflozin improved several CV risk factors in the CANVAS Program (Fig. 3) [16]. As expected, canagliflozin reduced HbA1c (mean [95% CI] − 0.58% [− 0.61 to − 0.56]), body weight (mean [95% CI] − 1.60 kg [− 1.70 to − 1.51]), systolic blood pressure (mean [95% CI] − 3.93 mm Hg [− 4.30 to − 3.56]), diastolic blood pressure (mean [95% CI] − 1.39 mm Hg [− 1.61 to − 1.17]), and increased high-density lipoprotein (HDL) cholesterol (mean [95% CI] +2.05 mg/dL [1.77 to 2.33]) [16]. An increase in LDL cholesterol in canagliflozin-treated patients was also found (mean [95% CI] +4.68 mg/dL [3.64 to 5.73]), while the LDL/HDL cholesterol ratio was not changed [16].

### Effects of canagliflozin on CV, mortality, and renal outcomes in the CANVAS Program

**Fig. 4:**
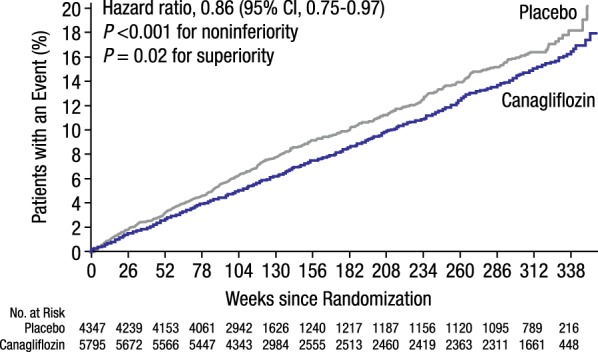
The CANVAS Program: CV death, nonfatal myocardial infarction, or nonfatal stroke. CV, cardiovascular; CI, confidence interval (Reprinted with permission from Neal [16])

The risk of an event in the primary composite outcome (MACE) was 14% lower with canagliflozin versus placebo (26.9 vs 31.5 participants with an event per 1000 patient-years; *P *< 0.001 for noninferiority and *P* = 0.02 for superiority; Fig. 4), without heterogeneity demonstrated between the CANVAS study and CANVAS-R [16]. Importantly, the effects of canagliflozin on the primary outcome were consistent across a range of different patient subgroups, except for subgroups by the use of diuretics and beta blockers at baseline, for which significant interactions were reported (*P* = 0.01 and *P* < 0.001, respectively). Based on these results, canagliflozin received indications from the FDA and Health Canada for reducing the risk of MACE in patients with T2DM and established CVD [24, 41].

Effect estimates suggest a benefit with canagliflozin treatment on all 3 components of the primary composite outcome compared with placebo (CV death [11.6 vs 12.8 per 1000 patient-years], nonfatal MI [9.7 vs 11.6 per 1000 patient-years], and nonfatal stroke [7.1 vs 8.4 per 1000 patient-years]), though individual effects did not reach statistical significance [16]. Effect estimates also suggested a benefit for all-cause mortality with canagliflozin versus placebo (17.3 vs 19.5 per 1000 patient-years), but this did not reach statistical significance [16].

**Fig. 5:**
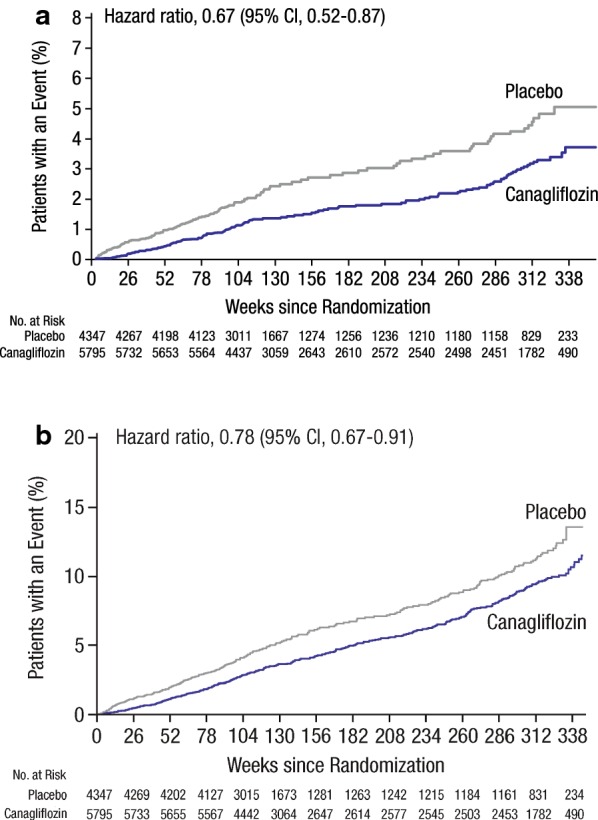
The CANVAS Program: **a** Hospitalization for HF and **b** CV death or hospitalization for HF. HF, heart failure; CV, cardiovascular; CI, confidence interval (Reprinted with permission from Rådholm [42])

Canagliflozin was also associated with improvements in heart failure (HF) outcomes. Patients randomized to canagliflozin experienced a 33% relative risk reduction of hospitalizations for HF (5.5 vs 8.7 per 1000 patient-years; Fig. 5a), as well as a 22% relative risk reduction of the composite outcome of CV death and hospitalization for HF (16.3 vs 20.8 per 1000 patient-years; Fig. 5b) and a 30% relative risk reduction for the composite of fatal HF or hospitalization for HF (6.4 vs 9.7 per 1000 patient-years) compared with placebo [16, 42]. Although results for the composite of CV death or hospitalization for HF were consistent between different subgroups, the presence of a baseline history of HF presented a borderline significant interaction, suggesting that perhaps the benefits of canagliflozin may be greater in those with a history of HF compared to those without [42]. In addition to baseline history of HF, other subgroups that may have a greater benefit from canagliflozin treatment on the composite of CV death or hospitalization for HF include patients with a body mass index ≥ 30 kg/m^2^, with HbA1c ≥ 8%, receiving diuretics, and not receiving metformin at baseline [42]. It should be noted that these HF analyses are exploratory analyses that warrant further evaluation.

**Fig. 6:**
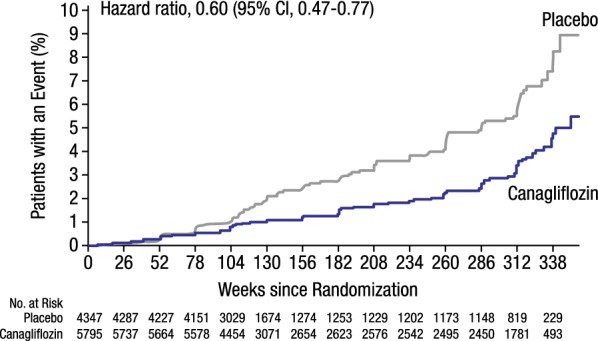
The CANVAS Program: composite of 40% reduction in eGFR, ESKD, or renal death. eGFR, estimated glomerular filtration rate; CI, confidence interval (Reprinted with permission from Neal [16])

Importantly, patients receiving canagliflozin also showed an improved renal profile compared with placebo: fewer patients experienced a progression of albuminuria (89.4 vs 128.7 per 1000 patient-years; 27% relative risk reduction) or new-onset albuminuria (100.4 vs 130.8 per 1000 patient-years; 20% relative risk reduction) [16, 43]. Patients randomized to canagliflozin had a 40% relative risk reduction in a composite renal outcome of 40% reduction in eGFR, end-stage kidney disease (ESKD), or renal death (5.5 vs 9.0 per 1000 patient-years; Fig. 6), and a 47% relative risk reduction in a composite of doubling of serum creatinine, ESKD, or renal death (1.5 vs 2.8 per 1000 patient-years) compared with placebo [16, 43]. Consistent effects of canagliflozin on renal outcomes were observed across patient subgroups by baseline renal function [44]. Overall, the effects of canagliflozin on renal function and renal outcomes in the CANVAS Program support a possible renoprotective effect in patients with T2DM [43]. The renal effects of canagliflozin have been further explored in the Canagliflozin and Renal Events in Diabetes with Established Nephropathy Clinical Evaluation (CREDENCE) trial of canagliflozin in patients with T2DM and chronic kidney disease, with overwhelming beneficial effects on both renal and CV events of canagliflozin compared to placebo [45].

The mechanism for the CV benefits seen with canagliflozin is unknown, but hypotheses include lowering of blood pressure, reduction of arterial stiffness, and amelioration of volume overload [16, 42]. Direct positive effects of canagliflozin on cardiac metabolism and enhanced cardiac efficiency may also contribute to the observed CV benefits [42]. Beneficial effects of canagliflozin on CV and renal outcomes were observed early on in the CANVAS Program and persisted over approximately 6 years of treatment, despite an increase in HbA1c over the course of the trial [16]. This suggests that canagliflozin may have HbA1c-independent effects on CV and renal outcomes.

### Adverse events of canagliflozin in the CANVAS Program and clinical considerations

Overall, serious adverse events occurred less frequently in those randomized to canagliflozin compared to placebo (104 vs 120 per 1000 patient-years; *P* = 0.04) [16]. There was no difference between the canagliflozin and placebo groups regarding frequency of discontinuation due to adverse events (35.5 vs 32.8 events per 1000 patient-years) [16]. Adverse events observed in the CANVAS Program were generally consistent with the known safety profile of canagliflozin and other SGLT2 inhibitors [46]. Adverse events that occurred at a higher frequency in participants randomized to canagliflozin included genitourinary infections, and osmotic diuresis; an increased risk of bone fractures with canagliflozin was observed in the CANVAS study, but not CANVAS-R, with no clear explanation for the heterogeneity [16]. An unanticipated increase in the risk of lower-limb amputation, at a low frequency of 3 excess events per 1000 patient-years and predominately at the level of the toe or metatarsal, was observed with canagliflozin. Importantly, canagliflozin was not associated with an increased risk of hypoglycemia, hyperkalemia, acute renal injury, diabetic ketoacidosis, pancreatitis, cancer, or venous thromboembolism.

#### Genitourinary infections

SGLT2 inhibitors increase the risk of genitourinary infections, presumably as a result of increased glucosuria [47]. In phase III clinical trials of canagliflozin, most genital mycotic infections occurred within the first 4 months of treatment in women and the first year of treatment in men, and few patients had more than 1 event [48]. The risk of genitourinary infections was higher among women with a prior history of vulvovaginitis and uncircumcised men [48]. In contrast, an increased risk of urinary tract infection (UTI) was not observed with canagliflozin versus placebo in the CANVAS Program (40 vs 37 per 1000 patient-years) [16]. On the other hand, canagliflozin was associated with a higher risk of mycotic genital infection in women (68.8 vs 17.5 per 1000 patient-years; *P* < 0.001) and infection of male genitalia (34.9 vs 10.8 per 1000 patient-years; *P* < 0.001), compared to placebo [16].

#### Osmotic diuresis and volume depletion

The hemodynamic effects of SGLT2 inhibitors, including reduced blood pressure and extracellular volume, are generally favorable effects and may explain, in part, the observed reductions in HF-related events and mortality. However, osmotic diuresis can lead to dehydration and may not be tolerated in all patients. Similar to previous trials of canagliflozin [49, 50], osmotic diuresis (34.5 vs 13.3 per 1000 patient-years; *P* < 0.001) and volume depletion (26.0 vs 18.5 per 1000 patient-years; *P* = 0.009) occurred more frequently with canagliflozin versus placebo in the CANVAS Program [16].

#### Risk of fracture

Low-trauma fracture (adjudicated by an endpoint adjudication committee) was the primary prespecified fracture outcome in the CANVAS Program, and significant heterogeneity was observed between the CANVAS study and CANVAS-R (*P* heterogeneity = 0.003); in the CANVAS study, an increased risk of low-trauma fracture was found to occur more frequently in those receiving canagliflozin compared to those receiving placebo (13.0 vs 8.3 per 1000 patient-years), but this was not observed in CANVAS-R (7.9 vs 10.3 per 1000 patient-years) [16]. Between-study heterogeneity was also observed in the secondary outcome of all fracture (adjudicated); patients in the CANVAS study had an increased risk of fracture with canagliflozin compared to placebo (16.9 vs 10.9 per 1000 patient-years), while no difference was observed in CANVAS-R (11.4 vs 13.2 per 1000 patient-years; *P* heterogeneity = 0.005) [16]. There is no clear explanation for the heterogeneity between the CANVAS study and CANVAS-R.

No increase in the risk of bone fracture was observed in a pooled analysis of non-CANVAS phase III clinical trials [51] or in the recent CREDENCE trial [45]. In a pooled analysis of phase III trials, canagliflozin was associated with a reduction in bone mineral density at the hip and an increase in bone turnover markers (e.g., osteocalcin) [52]. Similar results were observed in older patients (55–80 years of age), in whom canagliflozin was associated with a reduction in bone mineral density at the hip, but not the femoral neck, lumbar spine, or distal forearm, and an increase in osteocalcin [53]. These findings were consistent with the amount of weight loss observed with canagliflozin, and are not likely to represent deleterious effects on bone [52, 53].

A meta-analysis of all SGLT2 inhibitors found no overall increased risk of fracture with SGLT2 inhibitor use (odds ratio 1.14; 95% CI 0.86–1.52; *P* = 0.024) [54]. Of note, this analysis included data from the CANVAS Program and the EMPA-REG OUTCOME CVOT, which did not find an increased risk of fracture with empagliflozin [17]. In a recent analysis of 2 US commercial health care databases including more than 70 million patients comparing the effects of initiating canagliflozin or GLP-1 receptor agonists on the risk of fracture, no differences in the risk of fracture were reported [55].

Generally, fracture risk is higher in patients with diabetes who are older, have a history of CVD, lower eGFR, and diuretic use [56–59]. Therefore, an increased risk of fracture may be related to an increased risk of falls; the risk of falls is further increased by diabetes-related complications, such as concomitant use of antihypertensive agents that may induce orthostatic hypotension, diabetic neuropathy, and hypoglycemia [60, 61]. In the CANVAS Program and non-CANVAS studies of canagliflozin, the incidence of adverse events related to reported falls was low across treatment groups; however, these events were reported spontaneously, not actively collected, and were likely underreported [51].

#### Risk of amputation

In the CANVAS Program, an increased risk of lower-limb amputation was observed with canagliflozin compared with placebo (6.3 vs 3.4 per 1000 patient-years; *P* < 0.001), with 71% of amputations occurring at the toe or metatarsal [16]. A history of peripheral vascular disease and prior amputation were independent risk factors for amputation in the CANVAS Program, yet the relative risk was similar between canagliflozin and placebo across these subgroups [16]. No increase in the risk of amputation was observed in the canagliflozin phase III and IV study program of patients with T2DM and low CV risk [62], as well as in the recent CREDENCE trial of patients with T2DM and chronic kidney disease [45].

The mechanism by which canagliflozin raised the risk of amputation in the CANVAS Program remains unknown. Importantly, canagliflozin treatment does not appear to be associated with precipitating factors for amputation, including infections, gangrene, and diabetic foot ulcers [63]. Nevertheless, the FDA placed a boxed warning on the label of canagliflozin advising clinicians to carefully consider initiating canagliflozin in patients with risk factors for amputation and to monitor patients for signs and symptoms of sores or ulcers affecting the lower limbs [24].

It should be noted that systematic collection of data on amputations was not performed in EMPA-REG OUTCOME (amputation rate of 6.5 per 1000 patient-years with empagliflozin and placebo) [64]. No imbalance in the risk of amputation was observed in the overall population in the DECLARE-TIMI 58 CVOT of dapagliflozin, which was required to collect data on amputations, though data has not yet been reported for the secondary prevention cohort [65, 66]. The most recently approved SGLT2 inhibitor, ertugliflozin, carries a labeled warning for increased risk of lower-limb amputation (amputation rate of 6.8, 5.0, and 4.3 per 1000 patient-years with ertugliflozin 5 mg and 15 mg, and placebo, respectively, in phase III trials) [67].

Pharmacovigilance studies have further evaluated the risks of amputation with canagliflozin and other SGLT2 inhibitors. An analysis using the FDA Adverse Event Reporting System (FAERS) reviewed 66 cases of SGLT2 inhibitor-associated amputations. Canagliflozin was identified as a suspect or concomitant drug in 86% of cases (3.4 events per 1000 reports) [68]. Most amputations were of the toe, but 13 were above-ankle or limb amputations. Compared to non-SGLT2 inhibitor antihyperglycemic agents, the frequency of amputations was higher with canagliflozin with a proportional reporting ratio (PRR) of 5.33 (95% CI 4.04–7.04), while PRRs were not statistically different for dapagliflozin (PRR of 0.25 [95% CI 0.03–1.76]) and empagliflozin (PRR of 2.37 [95% CI 0.99–5.70]) [68]. Contrarily, an analysis using the World Health Organization (WHO) global database on individual case safety reports (VigiBase) did find a signal for increased risk of amputation of any type (PRR = 4.43 [95% CI 2.59–7.58]), the lower limb, and the toe with empagliflozin, and an increased risk of toe amputations only with dapagliflozin (PRR = 2.62 [95% CI 1.33–5.14]) [69]. Canagliflozin showed an increased risk of amputation of any type (PRR = 7.82 [95% CI 5.92–10.32]), the lower limb, the toe, and major amputation [69].

**Fig. 7:**
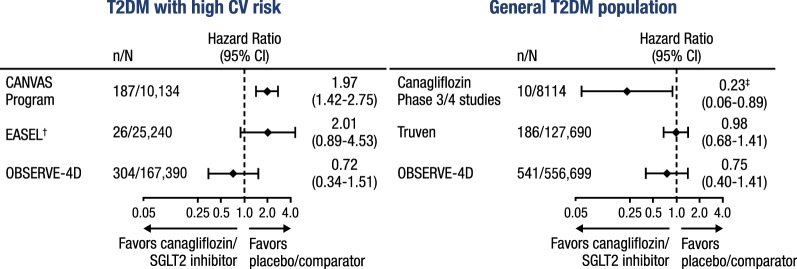
Risk of lower-limb amputation with SGLT2 inhibitor/canagliflozin. SGLT2, sodium glucose co-transporter 2; T2DM, type 2 diabetes mellitus; CV, cardiovascular. *CANVAS Program results are reported in the on-study population; Canagliflozin Phase 3/4 results are reported in the safety analysis set; Truven results are reported in the intent-to-treat population; EASEL and OBSERVE-4D results are reported in the on-treatment population. ^†^Comparison of SGLT2 inhibitor versus non-SGLT2 inhibitor. ^‡^Data are relative risk (95% CI)

In 3 recent observational, retrospective, new-user, real-world studies of patients with T2DM without high CV risk, patients treated with canagliflozin have shown a similar risk of lower-limb amputation compared to patients treated with other antihyperglycemic agents in intent-to-treat and on-treatment analyses (Fig. 7) [62, 70, 71]. One of these studies, OBSERVE-4D, included > 700,000 patients, negative controls outcomes to control for systematic error, and a confirmatory hospitalization for HF outcome [70]. One observational study in patients with established CVD found an increased risk of amputation with SGLT2 inhibitors compared to other antihyperglycemic therapies in an intent-to-treat analysis, but not an on-treatment analysis [72], while other studies have not shown an increased risk in this patient population [70, 71]. Limitations of observational studies include the inability to account for all potential confounders and the potential for miscoded claims. Some studies were limited by lower baseline CV risk and a younger patient population (mean age ~ 53 years), limiting the generalizability to older adults [62, 71].

## CV outcomes in the CANVAS Program, EMPA-REG OUTCOME, DECLARE-TIMI 58, LEADER, SUSTAIN-6, and Harmony Outcomes

In addition to the improved CV outcomes seen with canagliflozin, 5 other glucose-lowering agents have shown superior CV effects compared to placebo: SGLT2 inhibitors empagliflozin (EMPA-REG OUTCOME) and dapagliflozin (DECLARE-TIMI 58), and the GLP-1 receptor agonists liraglutide (LEADER), semaglutide (SUSTAIN-6), and albiglutide (Harmony Outcomes). The study designs and clinical findings of these CVOTs are discussed below and summarized in Table 1, but because of differences in study design and patient populations, the results of these studies cannot be directly compared.

**Table 1:** Key information for CVOTs of glucose-lowering agents with demonstrated CV benefit in patients with type 2 diabetes mellitus [16–19, 66, 76]

Class	Study name	Intervention	N of patients and median (max) follow-up	Inclusion criteria	Primary outcome(s)	Secondary findings
SGLT2 inhibitors	CANVAS Program (2017)	Canagliflozin 100 mg or 300 mg daily vs placebo	10,142 3.6 (6.5) years	≥ 30 years with established CVD (66%) or ≥ 50 years and ≥ 2 CV risk factors (34%)^a^	*MACE*^*b*^: Met criteria for noninferiority and superiority 14% RRR (overall) 18% RRR (secondary cohort)	33% ↓ HF hospitalizations No difference in ACM, CV death, or nonfatal stroke
	EMPA-REG OUTCOME (2015)	Empagliflozin 10 mg or 25 mg once daily vs placebo	7020 3.1 (4.0) years	≥ 18 years and established CVD (100%)^c^	*MACE*^*b*^: Met criteria for noninferiority and superiority 14% RRR	32% ↓ ACM 38% ↓ CV death 35% ↓ HF hospitalizations
	DECLARE-TIMI 58 (2018)	Dapagliflozin 10 mg daily vs placebo	17,160 4.2 years	≥ 40 years with established CVD (41%) or men ≥ 55 years and women ≥ 60 years with ≥ 1 CV risk factor (59%)^d^	*MACE*^*b*^: Met criteria for noninferiority but not superiority	27% ↓ HF hospitalizations No difference in CV death, nonfatal MI, nonfatal stroke, or ACM
					*CV death and hospitalization for HF:* Met criteria for noninferiority and superiority 14% RRR	
GLP-1 receptor agonists	LEADER (2016)	Liraglutide target dose of 1.8 mg daily vs placebo	9340 3.8 (4.5) years	≥ 50 years and established CVD (72.4%) or ≥ 60 years and ≥ 1 CV risk factor (27.6%)^e^	*MACE*^*b*^: Met criteria for noninferiority and superiority 13% RRR	15% ↓ ACM 22% ↓ CV death No difference in HF hospitalization
	SUSTAIN-6 (2016)	Semaglutide 0.5 mg, 1 mg once weekly vs placebo	3297 2.1 years	≥ 50 years and established CVD (83.0%) or ≥ 60 years and ≥ 1 CV risk factor (17.0%)^e^	*MACE*^*b*^: Met criteria for noninferiority and superiority 26% RRR	26% ↓ in nonfatal stroke No difference in ACM, CV death, or HF hospitalization
	Harmony Outcomes (2018)	Albiglutide 30–50 mg once weekly vs placebo	9463 1.6 (2.6) years	≥ 40 years and established CVD (100%)^f^	*MACE*^*b*^: Met criteria for noninferiority and superiority 22% RRR	22% ↓ in MACE and urgent coronary revascularization 25% ↓ in MI No difference in CV death, stroke, ACM, or CV death and hospitalization for HF

CVOT, cardiovascular outcomes trial; CV, cardiovascular; SGLT2, sodium glucose co-transporter-2; GLP-1, glucagon-like peptide-1; CVD, cardiovascular disease; MACE, major adverse cardiovascular event; RRR, relative risk reduction; HF, heart failure; ACM, all-cause mortality; MI, myocardial infarction; PVD, peripheral vascular disease; CAD, coronary artery disease; CKD, chronic kidney disease

^a^Includes patients with history of symptomatic atherosclerotic vascular disease (coronary, cerebrovascular, or peripheral), including stroke, MI, hospital admission for unstable angina, coronary artery bypass graft, percutaneous coronary intervention (with or without stenting), peripheral revascularization (angioplasty or surgery), symptomatic with documented hemodynamically-significant carotid or peripheral vascular disease, or amputation secondary to vascular disease. Risk factors include: duration of diabetes ≥ 10 years, systolic blood pressure > 140 mm Hg while receiving ≥ 1 antihypertensive agent, current smoking, microalbuminuria or macroalbuminuria, or high-density lipoprotein cholesterol levels of < 38.7 mg/dL (1 mmol/L)

^b^Composite outcome of CV death, nonfatal MI, or nonfatal stroke

^c^Includes patients with ≥ 1 of the following: history of MI or evidence of multivessel CAD (drug-naïve patients) or presence of significant stenosis; previous revascularization; combination of revascularization in 1 coronary artery and significant stenosis in another major coronary artery; evidence of single vessel CAD, ≥ 50% luminal narrowing during angiopathy not subsequently successfully revascularized with positive noninvasive stress test for ischemia and/or hospital discharge for unstable angina; unstable angina with evidence of single- or multi-vessel CAD; history of stroke; or occlusive peripheral artery disease documented by limb angioplasty, stenting, or bypass surgery, limb or foot amputation due to circulatory insufficiency, evidence of significant peripheral artery stenosis in 1 limb, or ankle brachial index < 0.9 in ≥ 1 ankle (patients on background therapy)

^d^Includes patients with clinically evident ischemic heart disease (documented MI, percutaneous coronary intervention, coronary artery bypass grafting, objective findings of coronary stenosis [≥ 50%] in ≥ 2 coronary artery territories [i.e., left anterior descending, ramus intermedius, left circumflex, right coronary artery] involving the main vessel, a major branch, or a bypass graft), cerebrovascular disease (documented ischemic stroke [known transient ischemic attack, primary intracerebral hemorrhage or sub-arachnoid hemorrhage do not qualify], carotid stenting, or endarterectomy), or peripheral artery disease (peripheral arterial intervention, stenting, or surgical revascularization; lower-extremity amputation as a result of peripheral arterial obstructive disease; or current symptoms of intermittent claudication and ankle/brachial index < 0.90 documented within last 12 months). Risk factors include: hypertension (blood pressure > 140/90 mm Hg at enrollment visit; patient must have both elevated systolic and diastolic blood pressure on both measurements), dyslipidemia (defined as low-density lipoprotein cholesterol levels > 130 mg/dL [3.36 mmol/L] or the use of lipid lower therapies), or the use of tobacco

^e^With ≥ 1 CV coexisting condition (coronary heart disease, cerebrovascular disease, PVD, CKD of stage 3 or greater or chronic HF of the New York Heart Association class II or III)

^f^Includes established disease of the coronary (MI, ≥ 50% stenosis in ≥ 1 coronary artery, or previous coronary revascularization), cerebrovascular (ischemic stroke, ≥ 50% carotid artery stenosis, or a previous carotid vascular procedure), or peripheral arterial circulation (intermittent claudication and an ankle to brachial index < 0.9, nontraumatic amputation, or a previous peripheral vascular procedure)

The CANVAS Program, DECLARE-TIMI 58, LEADER, and SUSTAIN-6 enrolled patients with and without established CVD at baseline, targeting both primary and secondary CVD prevention, while EMPA-REG OUTCOME and Harmony Outcomes enrolled only patients with established CVD at baseline. This is a key distinction because the number of CV events in primary prevention cohorts are typically numerically lower than those in secondary prevention cohorts.

In the EMPA-REG OUTCOME trial, empagliflozin met the primary composite CV endpoint of MACE (14% relative risk reduction; 37.4 vs 43.9 per 1000 patient-years; *P* < 0.001 for noninferiority and *P* = 0.04 for superiority) [17]. In the empagliflozin group, there were also lower rates of CV death, hospitalization for HF, all-cause mortality, and renal outcomes, including incident or worsening nephropathy [17, 73]. Based on the results of EMPA-REG OUTCOME, the FDA granted a change in the label for empagliflozin to include reduction in CV death [74].

In DECLARE-TIMI 58, dapagliflozin did not reduce the rate of the co-primary composite endpoint of MACE compared to placebo (22.6 vs 24.2 per 1000 patient-years), but did reduce the rate of the other co-primary endpoint, the composite of CV death and hospitalization for HF (17% relative risk reduction; 12.2 vs 14.7 per 1000 patient-years; *P* = 0.005); the reduction in the composite of CV death and hospitalization for HF was driven by a reduction in hospitalization for HF (6.2 vs 8.5 per 1000 patient-years) [66]. There were also lower rates of renal outcomes in the dapagliflozin group compared with placebo [66].

In a meta-analysis of the CANVAS Program, EMPA-REG OUTCOME, and DECLARE-TIMI 58, SGLT2 inhibitors were associated with a reduced risk of MACE outcomes, with an 11% relative risk reduction compared to placebo; benefits were only observed in patients with prior history of CV disease, not those with CV risk [75]. Benefits for MI, CV death, hospitalization for HF, all-cause mortality, and the composite of CV death and hospitalization for HF were also observed with SGLT2 inhibitors, although high between-study heterogeneity was observed for outcomes of CV death and all-cause mortality [75]. SGLT2 inhibitors were also associated with lower rates of renal outcomes compared with placebo [75]. As described in the prior paragraphs, despite presenting similar CV benefits, SGLT2 inhibitors may have different safety profiles, which clinicians should take into consideration.

In the LEADER trial, the primary CV composite endpoint (MACE), CV death and all-cause mortality, occurred in significantly fewer patients treated with liraglutide versus placebo (13% relative risk reduction; 34 vs 39 per 1000 patient-years; *P* = 0.01) [18]. The rate of death from any cause was also lower in the liraglutide group than in the placebo group [18]. As part of a non-prespecified analysis, a significant reduction in MI was also observed in liraglutide-treated patients, although there was no difference in fatal, nonfatal, and silent MI or in hospitalization for HF [18]. A lower incidence of nephropathy was observed with liraglutide [18].

Of the CVOTs that demonstrated a CV benefit, SUSTAIN-6 enrolled the fewest patients. The primary composite CV endpoint (MACE) occurred in significantly fewer patients with semaglutide versus placebo (relative risk reduction of 26%; 32.4 vs 44.4 per 1000 patient-years; *P* < 0.001 for noninferiority and *P* = 0.02 for superiority) [19]. The risk of nonfatal stroke and revascularization were reduced with semaglutide, but rates of CV death, all-cause mortality, and hospitalizations for HF were similar compared to placebo [19]. Rates of new or worsening nephropathy were lower with semaglutide, similar to improvements in renal outcomes observed in the CANVAS Program, EMPA-REG OUTCOME, DECLARE-TIMI 58, and LEADER [19].

In the Harmony Outcomes trial, albiglutide reduced the risk of the primary composite endpoint (MACE) compared with placebo (46 vs 59 per 1000 patient-years; *P* < 0.0001 for noninferiority and *P* = 0.0006 for superiority) [76]. The risk of fatal or nonfatal MI and the expanded composite outcome of CV death, MI, stroke, or urgent coronary revascularization for unstable angina was lower with albiglutide, but rates of CV death, fatal or nonfatal stroke, all-cause mortality, and the composite of CV death or hospitalization for HF were similar compared to placebo [76].

Because the CANVAS Program was composed of 2 clinical trials with different treatment allocation ratios and different follow-up, it is not possible to estimate the overall number needed to treat (NNT) to prevent 1 event in regard to the primary composite CV endpoint. However, for hypothesis-generating purposes, a NNT based on the annualized rate (i.e., up to 3 years) can be calculated for trials that showed superiority for MACE. When comparing similar populations investigated in CVOTs discussed above (primary and secondary prevention), the NNT (primary CV endpoint) in LEADER, SUSTAIN-6, and the CANVAS Program was 67, 28, and 72, respectively. When comparing the secondary prevention cohort of the CANVAS Program with EMPA-REG OUTCOME and Harmony Outcomes, the NNT (primary composite CV endpoint) was 46, 51, and 26, respectively. The annualized rates for LEADER and SUSTAIN-6 were not reported in the different cohorts and NNT was not calculated for DECLARE as it did not show superiority for MACE. Of note, additional analyses have shown that canagliflozin along with empagliflozin and liraglutide were superior to placebo in regard to improving CV outcomes [77].

## Guideline recommendations

Evidence from recent CVOTs demonstrating, for the first time, that select antihyperglycemic therapies can reduce CVD risk and mortality has greatly impacted clinical practice guidelines. Selection of antihyperglycemic therapies is no longer based solely on their ability to lower HbA1c, but also their effects on CV risks. This change is reflected in recent clinical practice guidelines for the management of T2DM and recommendations for the management of HF.

The 2018 Consensus Report released by the American Diabetes Association (ADA) and the European Association for the Study of Diabetes as well as the most recent 2019 ADA Standards of Medical Care in Diabetes guidelines continue to recommend metformin as first-line glucose-lowering therapy for most patients with T2DM; however, it is now recommended that clinicians consider the patient’s underlying CV risk when selecting an additional glucose-lowering agent to use in combination with metformin [78, 79]. The ADA guidelines recommend adding empagliflozin, liraglutide, or canagliflozin in patients with established CVD [78]. Similarly, the 2018 American Association of Clinical Endocrinologists (AACE)/American College of Endocrinology (ACE) Comprehensive Type 2 Diabetes Management Algorithm also recommends GLP-1 receptor agonists and SGLT2 inhibitors as preferred add-on agents to metformin and lifestyle with no preference for a specific agent within each class [80].

SGLT2 inhibitors and GLP-1 receptor agonists as cardioprotective agents have also received interest from cardiology societies. Notably, the 2017 American College of Cardiology (ACC) Expert Decision Pathway for Optimization of Heart Failure Treatment included an “intermediate” recommendation to consider SGLT2 inhibitors in patients with HF and diabetes [81]. The ACC has also hosted a multidisciplinary round table of experts to weigh in on how clinicians should navigate the use of these medications, which are now more likely to be prescribed by non-diabetologists. In order to provide guidance for cardiologists, the ACC is currently developing an Expert Consensus Decision Pathway document to provide guidance for CV clinicians on how and when to prescribe SGLT2 inhibitors and GLP-1 receptor agonists. Additionally, the European Society of Cardiology (ESC), in collaboration with other CVD prevention societies, put forth recommendations in 2016 that included the use of SGLT2 inhibitors in patients with diabetes and established CVD [82]. The 2017 National Lipid Association guidelines for patients with T2DM also suggest using antihyperglycemic agents with favorable effects on CVD in patients with T2DM and coronary artery disease and/or HF [83]. Although the CANVAS Program and EMPA-REG OUTCOME showed favorable effects on HF, the overall number of events was smaller than that typically seen in HF trials. Clinical trials specifically powered to detect the effects of SGLT2 inhibitors in patients with HF, with and without T2DM, are currently ongoing [84, 85].

## Conclusion

The CANVAS Program demonstrated that canagliflozin improves several cardiometabolic risk factors and reduces major CV events in patients with T2DM. Beneficial effects on HF and renal outcomes were also observed. As with any new pharmacological therapy, canagliflozin is not without side-effects; therefore, benefits and risks must be carefully considered by the clinician. Ongoing clinical trials of SGLT2 inhibitors, including canagliflozin, and mechanistic studies will hopefully shed light on the adverse events identified in the CANVAS Program (i.e., fractures and amputations).

In conclusion, canagliflozin, along with other antihyperglycemic agents with proven beneficial CV effects (i.e., empagliflozin, dapagliflozin, liraglutide, semaglutide, and albiglutide), represents a new opportunity for the diabetologist and cardiologist, in the setting of a multidisciplinary approach, to concomitantly improve glycemic control and reduce the risk of CV events in patients with T2DM.

## Data Availability

Not applicable.

## Abbreviations

SGLT2: sodium glucose co-transporter 2
FDA: US Food and Drug Administration
T2DM: type 2 diabetes mellitus
HbA1c: glycated hemoglobin
CANVAS: CANagliflozin cardioVascular Assessment Study
CANVAS-R: CANagliflozin cardioVascular Assessment Study–Renal
CVOT: cardiovascular outcomes trial
CV: cardiovascular
CVD: cardiovascular disease
MACE: major adverse cardiovascular events
MI: myocardial infarction
GLP-1: glucagon-like peptide-1
RT_G_: renal threshold for glucose
UGE: urinary glucose excretion
eGFR: estimated glomerular filtration rate
LDL: low-density lipoprotein
HR: hazard ratio
CI: confidence interval
HDL: high-density lipoprotein
HF: heart failure
ESKD: end-stage kidney disease
CREDENCE: Canagliflozin and Renal Events in Diabetes with Established Nephropathy Clinical Evaluation
UTI: urinary tract infection
PVD: peripheral vascular disease
FAERS: FDA Adverse Event Reporting System
PRR: proportional reporting ratio
WHO: World Health Organization
NNT: number needed to treat
ADA: American Diabetes Association
AACE: American Association of Clinical Endocrinologists
ACE: American College of Endocrinology
ACC: American College of Cardiology
ESC: European Society of Cardiology
